# Surgical smoke in hysteroscopic surgery: Does it really matter in COVID-19 times?

**Published:** 2020-05-07

**Authors:** M Farrugia

**Affiliations:** Spencer Private Hospitals, Kent, United Kingdom.

We live in unusual times. If concern about aerosol spread had never crossed the average person’s mind, it certainly has now!

In open surgery and endoscopy, we have concerned ourselves with splashes and aerosols for many years, and there is a body of work that is both reassuring and concerning to those who need or wish to carry out procedures in these challenging times.

Open surgery, laparoscopy and hysteroscopy are carried out in different environments, and on a variety of types of human tissue, using a range of energy sources and instrumentation. Energy sources, particularly electrosurgery and laser, generate a wide range of chemical compounds in the air during open procedures, in carbon dioxide during laparoscopy and in a liquid distension medium in hysteroscopy.

The initial concern with surgical smoke, diathermy plume, mist, vapour, aerosol (or whatever name you may wish to call it) was of its composition, potential cytotoxicity and teratogenicity. Sagar et al. (1996) looked at the chemical composition of an electrosurgical plume on porcine liver, and identified over 100 volatile compounds, of which benzene and acrylonitrile are potential carcinogens. These studies were later replicated on various human tissue types and later in vivo.

Awareness of potential infectivity and malignant risk from cell fragments following the use of lasers, electrosurgery and powered surgical instruments preceded the concern of what type of hydrocarbons were in the surgical plume. Human papilloma virus (HPV) DNA was identified in the plume after treatment of human warts treated with a CO_2_ laser and electrocoagulation. Not surprisingly, cervical intra-epithelial neoplasia treated using laser and electrosurgery results in the ejection of HPV DNA in the surgical plume (Garden et al., 1988). Viable melanoma cells have also been recovered from the smoke after the use of electrosurgery, as well as various tumour cell lines following the use of electrosurgical cutting, coagulation and ultrasonic scalpels (Fletcher et al., 1999).

Once the concept of how these volatile gases, non-volatile compounds and cellular debris are produced is understood, one can apply them to other forms of surgical interventions. Hysteroscopy and hysteroscopic electrosurgery takes place in an ‘underwater’ environment. Fulguration or spray coagulation, commonly employed in open or laparoscopic surgery, cannot occur in a liquid medium. Steam and smoke generated are cooled down instantaneously to the temperature of the distension medium. Water soluble particles in smoke bubbles will dissolve in the distension medium. Therefore, hysteroscopic surgery with electrical devices, laser and mechanical energy operate in a unique environment.

Bubbles have always preoccupied the hysteroscopist, mainly due to the risk of an embolism and its consequences. From a scientific point of view, a bubble is a region of air or vapour trapped by a thin film. True bubbles have two surfaces so what we see in modern hysteroscopy are not strictly speaking bubbles, but cavities. A cavity is a hole in a liquid filled with vapour (Atkins, 1987) and possesses only one surface. This is important because small cavities, say smaller than 10μm in diameter collapse due to their high internal pressure (created by the surface tension of the distension medium). Bigger cavities seen at hysteroscopy cool down, steam condenses and soluble components dissolve, as described above. The only true bubbles observed personally were created during CO_2_ hysteroscopy with blood in the uterine cavity (this was over two decades ago). There are no bubbles generated during hysteroscopy that go on to be released into the atmosphere, eventually popping and releasing its contents.

Gaseous breakdown products from human uterine tissue during hysteroscopic electrosurgery are very different to those generated in an oxygen atmosphere in open surgery and a CO_2_ atmosphere in laparoscopy. The products of cellular breakdown during hysteroscopic electrosurgery were characterised as part of my PhD work (Farrugia, 2009) and identified the quantity and composition of gases released as well as insoluble particles. Low molecular weight molecules such as hydrogen, oxygen, nitrogen, carbon monoxide and dioxide, as well as hydrocarbons such as ethyne and ethene, dissipate rapidly in blood. Particles ranging from 0.1μm to granules up to 2mm were released and measured, averaging diameter 0.128μm in diameter for monopolar instruments and 0.254μm for bipolar instruments (Farrugia et al., 2009) . They are on average smaller than those generated by laser vaporisation (median diameter 0.31μm), but unlike the latter have no aerodynamic significance as they are washed away by a liquid distension medium.

From a patient’s perspective, these gases and particles are of significance because of their potential clinical effects, which are still under investigation. Gas emboli were first shown to occur in monopolar surgery by Bloomstone et al. (2002). However, small particles generated during hysteroscopy have been shown to be echogenic, and can mimic gas emboli (Farrugia, 2009). There is more work that needs to be done on this topic, as tissue fragments will also be released by mechanical devices and have the potential of resulting in clinical situations mimicking an embolus.

For surgeons working in today’s environment, it is important to understand that although hysteroscopic procedures are not high-risk aerosol generating interventions, the necessary precautions to prevent infection are still important. Hysteroscopists should ensure that negative pressure on the outflow channel should be applied, carrying away any gaseous and particulate surgical by-products into sealed containers.

These recommendations are echoed by the ESGE guidelines (2020) and the GCH Committee consensus statement on hysteroscopic surgery during the COVID-19 pandemic (Carugno et al., 2020). Patients requiring hysteroscopy, particularly those suspected of endometrial cancer, should not be denied this procedure, particularly if carried out in an office setting. This will ensure minimal exposure of the patient and staff, without delaying diagnosis and treatment. The long-term health impact of patients not seeking appropriate medical care to avoid exposure to COVID-19 is already noticeable in general population mortality rates (Centre for Evidence Based Medicine, 2020). We should make an effort to reduce this to a minimum by playing our part as hysteroscopists in these difficult times.
